# Resistance to fosfomycin is increasing and is significantly associated with extended-spectrum β-lactamase-production in urinary isolates of *Escherichia coli*

**DOI:** 10.1007/s00430-022-00749-2

**Published:** 2022-09-03

**Authors:** Esther Ríos, María del Carmen López Diaz, Esther Culebras, Iciar Rodríguez-Avial, Carmen Rodríguez-Avial

**Affiliations:** 1 Servicio de Microbiología, Hospital Clínico San Carlos, C/ Profesor Martín Lagos, s/n., 28040 Madrid, Spain; 2 Departamento de Medicina (Microbiología), Facultad de Medicina, Universidad Complutense de Madrid, Pza. Ramón y Cajal, s/n, 28040 Madrid, Spain

**Keywords:** Antimicrobial resistance, Fosfomycin, *Escherichia coli*, ESBL

## Abstract

Fosfomycin has become a therapeutic option in urinary tract infections. Our objective was to evaluate the in vitro activity of fosfomycin against *Escherichia coli* isolated from urine samples in 2013, 2018 and 2021. We also determined a putative association between fosfomycin resistance and extended-spectrum β-lactamases (ESBL) production. Fosfomycin activity was evaluated against 7367, 8128 and 5072 *Escherichia coli* urinary isolates in 2013, 2018 and 2021, respectively. We compare the prevalence of fosfomycin-resistant strains among the ESBL- and non-ESBL-producing isolates. MICs of fosfomycin, cefotaxime, and cefotaxime-clavulanate were determined by a microdilution method. 302 ESBL-producers were selected to determine MICs of fosfomycin by agar dilution and genes encoding ESBLs were detected by PCR. Among the total of ESBL-producing strains, 14.3%, 20.8% and 20% were resistant to fosfomycin in 2013, 2018 and 2021, respectively, whereas fosfomycin resistance in non-ESBL producers was 3.5%, 4.05% and 5.53% for each year (*P* ≤ 0.001). In the 302 selected ESBL-producing isolates, CTX-M was the main ESBL (228 isolates), being 50.7% CTX-M-15. Resistance to fosfomycin among these ESBL-producing strains was associated (*P* = 0.049) with isolates that produced the CTX-M type. Our data show that fosfomycin resistance is increasing in *Escherichia coli* urinary isolates and it is related to ESBL-production. A follow-up of fosfomycin resistance is required.

## Introduction

Acquired resistance to β-lactams is mostly mediated by extended-spectrum β-lactamases (ESBLs). Recently, a dramatic increase of CTX-M enzymes has been observed, mainly associated with *Escherichia coli* urinary isolates [1]. These strains usually possess plasmids that carry genes conferring resistance to multiple antibiotic classes. These multidrug-resistance plasmids have become an increasing concern due to their role in the spread of ESBLs β-lactamases, among *E. coli* [2]. As a result, therapeutic options against these β-lactam-resistant *E. coli* infections are extremely limited.

Fosfomycin is a traditional antimicrobial agent with broad-spectrum bactericidal activity. It has been used as an alternative for treatment of uncomplicated lower urinary tract infections (UTI) caused, mainly, by *E. coli* [3]. The recent growing prevalence of ESBL-producing Enterobacterales and fluoroquinolone resistant *E. coli* has rekindled interest in fosfomycin as a therapeutic agent in many countries [4, 5]. Several mechanisms of fosfomycin resistance have been reported in *E. coli*: chromosomal mutations in the target gene (*murA*) or in fosfomycin transporter genes (*glpT* and *uhpT*), and the acquisition of plasmid-mediated *fos* genes that inactivate fosfomycin [6].

In the present study, we analyzed the prevalence of fosfomycin resistance in *E. coli* urinary isolates. We also determined putative association between fosfomycin resistance and ESBL production.

## Materials and methods

The study includes all *E. coli* strains isolated from urine samples at the Hospital Clínico San Carlos (Madrid, Spain) in 2013, 2018 and 2021. Only one isolate per patient was included.

Initially, bacterial identification and antimicrobial susceptibility tests were conducted using MicroScan panels (Beckman Coulter, Brea, CA, USA). The in vitro activity of fosfomycin, ceftazidime, cefotaxime, ceftriaxone, cefotaxime-clavulanate, ceftazidime-clavulanate and nitrofurantoin was evaluated by using the microdilution method (MicroScan panels).

A representative sample of 302 ESBL-producing *E. coli* isolates was selected to confirm the MIC of fosfomycin by the agar dilution method and ESBL production. Fosfomycin susceptibility was performed by the reference agar dilution method, containing glucose-6-phosphate (Sigma-Aldrich, St. Louis, MO), according to Clinical and Laboratory Standards Institute (CLSI) guidelines [7]. ESBL production was confirmed phenotypically, using both cefotaxime and ceftazidime alone or in combination with clavulanic acid, following CLSI guidelines [7].

Antibiotic susceptibility was interpreted according to the European Committee on Antimicrobial Susceptibility Testing (EUCAST) criteria and oral breakpoints (S ≤ 32 mg/L; R > 32 mg/L) was considered for fosfomycin susceptibility [8]. *E. coli* ATCC 25922, *E. coli* ATCC 35218 and *P. aeruginosa* ATCC 27853 were used as control strains in susceptibility assays.

The genes encoding ESBLs were detected by PCR in the 302 ESBL-producing *E. coli* strains as described previously [9]. Isolates from our collection that were previously characterized were used as controls in the PCR assays.

The proportion of isolates with different MICs or resistance profiles using breakpoints by EUCAST was compared by Fisher’s exact test. In correlations between pairs of variables, significance was defined as *P* ≤ 0.05.

## Results

A total of 20,577 *E. coli* isolates from urine samples were collected over the three years: 7367 strains in 2013, 8128 strains in 2018 and 5072 strains in 2021. Table 1 shows the percentages of fosfomycin resistance (total, ESBL and non-ESBL producers). During these years of study, the fosfomycin resistance rates were 4.3%, 5.45% and 6.6%, respectively.

**Table 1:** Resistance of *Escherichia coli* urinary isolates to fosfomycin

	2013 (*N* = 7367)	2018 (*N* = 8128)	2021 (*N* = 5072)
	No. (%)	No. (%)	No. (%)
Total isolates	317 (4.3)	443 (5.45)	334 (6.6)
ESBL-producers	78 (14.3)^a^	141 (20.8)^a^	74 (20)^a^
non-ESBL producers	239 (3.5)^a^	302 (4.05)^a^	260 (5.5)^a^

^a^*P* ≤ 0.001

Among the total of ESBL-producing strains, 14.3%, 20.8% and 20% were resistant to fosfomycin in 2013, 2018 and 2021, respectively, whereas fosfomycin resistance in non-ESBL-producers was 3.5%, 4.05% and 5.53% for each year. There were significant differences in the fosfomycin resistance rates when ESBL-producers were compared to non-ESBL-producers (*P* ≤ 0.001).

Of the 302 selected ESBL-producing *E. coli*, *bla*CTX-M was the main gene detected (228 isolates, 75.6%) followed by *bla*SHV (14.2%) and *bla*TEM (8.3%). Among the 228 CTX-M ESBL-producing *E. coli* isolates, 117 (51.3%) carried *bla* CTX-M-15 and 111 (48.7%) *bla* CTX-M group 9. Resistance to fosfomycin was 15.3%, being MIC50 and MIC90 values 1 mg/L and 64 mg/L, respectively. The fosfomycin resistance rate was also evaluated according to ESBL-type (Table 2), being significantly higher among CTX-M-producers than among non-CTX-M producers (18.2% vs. 8.1%, *P* = 0.049). Moreover, the MIC90 of fosfomycin against CTX-M-producing *E. coli* strains was higher compared to those against non-CTX-producers (MIC90 = 64 mg/L vs. MIC90 = 8 mg/L).

**Table 2:** In vitro susceptibility to fosfomycin of 302 *Escherichia coli,* related to the ESBL- type produced

Enzyme	MIC_50_ (mg/L)	MIC_90_ (mg/L)	Range (mg/L)	Resistance^a^ (%)	*P* value
CTX-M (*n* = 228)	1	64	≤ 0.5–256	40 (18.2)	0.049
Non CTX-M (*n* = 74)	1	8	≤ 0.5–128	6 (8.1)	

^a^Based on EUCAST-susceptibility breakpoints

## Discussion

Resistance to fosfomycin among our ESBL-producing strains was 14.3%, 20.8% and 20% in 2013, 2018 and 2021, respectively. Our data are comparable to those obtained by previous studies using EUCAST criteria [10–12]. Nevertheless, the evaluation of fosfomycin activity depends on susceptibility breakpoints used. EUCAST breakpoints are stricter than those of CLSI and this fact should be taken into account to compare with other surveys. Falagas et al*.* [13], in a review of 17 studies, found that 96.8% of ESBL-producing *E. coli* isolates were susceptibility to fosfomycin. Eleven of the 17 included studies used criteria corresponding to the CLSI breakpoints for *E coli* urinary isolates. Using a MIC susceptibility breakpoint of 64 mg/L or less according to CLSI, our study showed a similar percentage. Among the 302 selected ESBL producers, 275 (91.1%) were susceptible to fosfomycin, but then using EUCAST criteria, this rate was reduced to 84.7%.

In the current study, there are technical limitations in the in vitro susceptibility testing. All strains (recovered during 2013, 2018 and 2021) were tested for fosfomycin resistance by the broth microdilution method using MicroScan panels. However, only in 302 ESBL-producing *E. coli* isolates the fosfomycin MIC was confirmed by the reference agar dilution method according to CLSI guidelines. The number of selected strains was representative of the three study periods (2011-2018-2021) and the same rate of fosfomycin resistance was observed by both microdilution and agar dilution methods (15.3%, using EUCAST criteria).

In Spain, there is a trend towards increased fosfomycin resistance in ESBL-producing *E. coli* isolated from UTI [14]. A significant rise was also previously observed in our laboratory between 2005 (4.4%) and 2009 (11.4%), which in 2011 reached 14% [15]. Key factors related to this increased fosfomycin resistance could be the rapid growth in community use of fosfomycin in Spain in recent years [14].

This study demonstrated that resistance to fosfomycin among CTX-M- producing *E. coli* strains was higher than those producing SHV or TEM. The difference was statistically significant (*P* = 0.049) and it makes us think of the interest of studying the type of resistance of these strains. The main type of resistance to fosfomycin appears to be chromosome mediated rather than plasmid mediated [16]. However, two novel plasmid-mediated fosfomycin-modifying enzymes, FosA3 and FosC2, were recently identified in CTX-M-producing *E. coli* in Japan [17]. In a recent review, the authors assert that the CTX-M type β-lactamases have overall become the most prevalent ESBLs worldwide [1]. Transferable plasmids carrying *fosA3* or *fosC2* might accelerate the dissemination of fosfomycin resistance around the world. The *bla*CTX-M genes often coexist with other genes such as *fosA3*, which confers resistance to fosfomycin [18].

In conclusion, our results demonstrate that fosfomycin retains good activity against *E. coli* urinary isolates, suggesting that it may be a therapeutic option for the treatment of uncomplicated cystitis. However, an increasing resistance trend is observed for fosfomycin in ESBL-producing *E. coli* isolated from UTI, a follow-up of this resistance is required.

## Data Availability

All data generated or analyzed during this study are included in this published article.

## References

[1] D'Andrea MM, Arena F, Pallecchi L, Rossolini GM (2013). CTX-M-type beta-lactamases: a successful story of antibiotic resistance. Int J Med Microbiol.

[2] Woodford N, Carattoli A, Karisik E, Underwood A, Ellington MJ, Livermore DM (2009). Complete nucleotide sequences of plasmids pEK204, pEK499, and pEK516, encoding CTX-M enzymes in three major *Escherichia coli* lineages from the United Kingdom, all belonging to the international O25:H4-ST131 clone. Antimicrob Agents Chemother.

[3] Gupta K, Hooton TM, Naber KG, Wullt B, Colgan R, Miller LG (2011). International clinical practice guidelines for the treatment of acute uncomplicated cystitis and pyelonephritis in women: a 2010 update by the Infectious Diseases Society of America and the European Society for Microbiology and Infectious Diseases. Clin Infect Dis.

[4] Falagas ME, Maraki S, Karageorgopoulos DE, Kastoris AC, Mavromanolakis E, Samonis G (2010). Antimicrobial susceptibility of multidrug-resistant (MDR) and extensively drug-resistant (XDR) *Enterobacteriaceae* isolates to fosfomycin. Int J Antimicrob Agents.

[5] Oteo J, Perez-Vazquez M, Campos J (2010). Extended-spectrum [beta]-lactamase producing *Escherichia coli*: changing epidemiology and clinical impact. Curr Opin Infect Dis.

[6] Castañeda-García A, Blázquez J, Rodríguez-Rojas A (2013). Molecular mechanisms and clinical impact of acquired and intrinsic fosfomycin resistance. Antibiotics.

[7] CLSI. (2009) Performance standards for antimicrobial susceptibility testing. Nineteenth informational supplement. 29(3): CLSI document M100-S19. CLSI, Wayne, PA.3.

[8] Testing EUCAST. (2013) Break-point tables for interpretation of MICs and zone diameters. Version 3.1. European Committee on Antimicrobial Susceptibility Testing. https://www.eucast.org/fileadmin/src/media/PDFs/EUCAST_files/Breakpoint_tables/Breakpoint_table_v_3.1.pdf

[9] Coque TM, Oliver A, Perez-Diaz JC, Baquero F, Canton R (2002). Genes encoding TEM-4, SHV-2, and CTX-M-10 extended-spectrum beta-lactamases are carried by multiple *Klebsiella pneumoniae* clones in a single hospital (Madrid, 1989 to 2000). Antimicrob Agents Chemother.

[10] Kansak N, Arıcı N, Adaleti R, Nakipoglu Y, Aksaray S (2021). Rapid detection of fosfomycin resistance in *Escherichia coli* and *Klebsiella* spp. strains isolated from urinary tract infections. J Microbiol Methods.

[11] Oteo J, Orden B, Bautista V, Cuevas O, Arroyo M, Martínez-Ruiz R (2009). CTX-M-15-producing urinary *Escherichia coli* O25b-ST131-phylogroup B2 has acquired resistance to fosfomycin. J Antimicrob Chemother.

[12] Loras C, Constança Mendes A, Peixe L, Novais Â, Alós JI (2020). *Escherichia coli* resistant to fosfomycin from urinary tract infections: detection of the *fosA3* gene in Spain. J Glob Antimicrob Resist.

[13] Falagas ME, Kastoris AC, Kapaskelis AM, Karageorgopoulos DE (2010). Fosfomycin for the treatment of multidrug-resistant, including extended-spectrum β- lactamase producing, Enterobacteriaceae infections: a systematic review. Lancet Infect Dis.

[14] Oteo J, Bautista V, Lara N, Cuevas O, Arroyo M, Fernandez S (2010). Parallel increase in community use of fosfomycin and resistance to fosfomycin in extended-spectrum beta-lactamase (ESBL)-producing *Escherichia coli*. J Antimicrob Chemother.

[15] Rodriguez-Avial C, Rodriguez-Avial I, Hernandez E, Picazo JJ (2013). Increasing prevalence of fosfomycin resistance in extended-spectrum-beta-lactamase-producing *Escherichia coli* urinary isolates (2005–2009-2011). Rev Esp Quimioter.

[16] Takahata S, Ida T, Hiraishi T, Sakakibara S, Maebashi K, Terada S (2010). Molecular mechanisms of fosfomycin resistance in clinical isolates of *Escherichia col*i. Int J Antimicrob Agents.

[17] Wachino J, Yamane K, Suzuki S, Kimura K, Arakawa Y (2010). Prevalence of fosfomycin resistance among CTX-M-producing *Escherichia coli* clinical isolates in Japan and identification of novel plasmid-mediated fosfomycin-modifying enzymes. Antimicrob Agents Chemother.

[18] Hou J, Huang X, Deng Y, He L, Yang T, Zeng Z (2012). Dissemination of the fosfomycin resistance gene fosA3 with CTX-M beta-lactamase genes and *rmt*B carried on IncFII plasmids among *Escherichia coli* isolates from pets in China. Antimicrob Agents Chemother.

